# Continuous Flow of Upper Labrador Sea Water around Cape Hatteras

**DOI:** 10.1038/s41598-018-22758-z

**Published:** 2018-03-14

**Authors:** Magdalena Andres, Mike Muglia, Frank Bahr, John Bane

**Affiliations:** 1 Woods Hole Oceanographic Institution, Woods Hole, MA 02543 USA; 2 University of North Carolina Coastal Studies Institute, Wanchese, NC 27981 USA; 3 University of North Carolina, Chapel Hill, NC 27599 USA

## Abstract

Six velocity sections straddling Cape Hatteras show a deep counterflow rounding the Cape wedged beneath the poleward flowing Gulf Stream and the continental slope. This counterflow is likely the upper part of the equatorward-flowing Deep Western Boundary Current (DWBC). Hydrographic data suggest that the equatorward flow sampled by the shipboard 38 kHz ADCP comprises the Upper Labrador Sea Water (ULSW) layer and top of the Classical Labrador Sea Water (CLSW) layer. Continuous DWBC flow around the Cape implied by the closely-spaced velocity sections here is also corroborated by the trajectory of an Argo float. These findings contrast with previous studies based on floats and tracers in which the lightest DWBC constituents did not follow the boundary to cross under the Gulf Stream at Cape Hatteras but were diverted into the interior as the DWBC encountered the Gulf Stream in the crossover region. Additionally, our six quasi-synoptic velocity sections confirm that the Gulf Stream intensified markedly at that time as it approached the separation point and flowed into deeper waters. Downstream increases were observed not only in the poleward transport across the sections but also in the current’s maximum speed.

## Introduction

**Figure 1:**
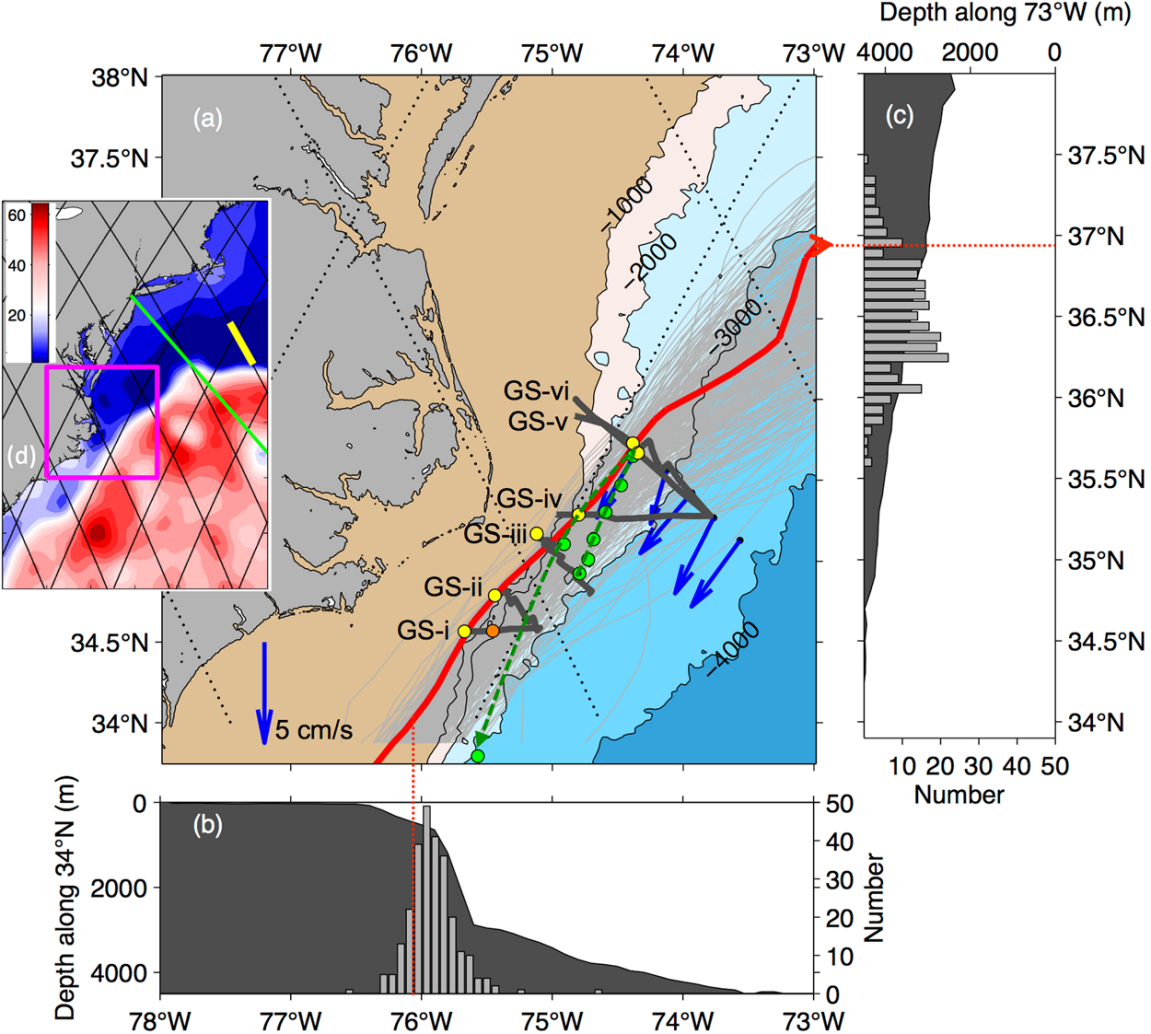
Panel (a) bathymetry around Cape Hatteras (shaded) showing six shipboard ADCP Gulf Stream crossings (dark gray), the ensemble of Gulf Stream paths from monthly-mean 25-cm SSH contours (light gray), the Gulf Stream path during the cruise estimated from the daily altimetry product on 20 April 2017 (red), and an Argo float trajectory (green). Also shown are locations of near-surface (yellow) and one sub-surface (orange) velocity maxima observed on each Gulf Stream crossing. Blue vectors indicate historical mean near-bottom DWBC flow observed with SYNOP moorings^5^. In the velocity sections examined here, the directions of the Gulf Stream velocity maxima at each ADCP crossing are generally well-aligned with the red SSH contour and the ULSW flows are generally well-aligned with the bathymetry. Panel (b) histogram of the longitude of “attached” Gulf Stream paths at 34°N superimposed on bottom topography. Panel (c) analogous histogram for the latitude of the “free” Gulf Stream at 73°W superimposed on bottom topography. Panel (d) regional map with the mapped SSH during the cruise period contoured (cm). The magenta box highlights the area shown in panel (a) and the Line W mooring section (yellow)^17^ and the Oleander Line (green)^24^ are also plotted. The maps in (a) and (d) were generated using Matlab 2014b (Mathworks, Inc.).

The “attached” Gulf Stream—the western boundary current of the wind-driven North Atlantic subtropical gyre—follows the shelf break of the southeastern United States to Cape Hatteras, where it separates abruptly from the continental margin and proceeds to the northeast (Fig. 1). Gulf Stream path deflections and meanders do occur there^1,2^, but are comparatively small amplitude. Downstream of the Stream’s time-varying separation point near ~35.5°N^3^, path variability is greatly enhanced as the Stream detaches from the steep continental slope and flows into deeper waters^4^.

Near Cape Hatteras, the poleward-flowing Gulf Stream encounters and crosses over the Deep Western Boundary Current (DWBC) which carries waters of the Atlantic Meridional Overturning Circulation’s (AMOC’s) cold limb equatorward^5^ (blue arrows in Fig. 1a). Both interior and DWBC pathways are thought to play a role in the export of AMOC cold limb waters from the subpolar to the subtropical gyre on its equatorward route^6^. The relative importance of these pathways, their variability, and the mechanisms that drive exchange of these deep waters between the boundary and interior have been the focus of both observational and modeling studies^7–9^.

Here we discuss a set of recent, high-horizontal-resolution observations of the flows around Cape Hatteras where the DWBC and Gulf Stream have been observed to interact with one another and with the bathymetry^5,10–12^. Previous studies have shown that the Gulf Stream’s detachment latitude may be influenced by the strength of the DWBC here^13^. In addition, those DWBC waters that pass under the separating Gulf Stream in the broad “crossover region” around Cape Hatteras are thought to move to deeper isobaths, conserving their potential vorticity as they continue equatorward along the boundary^14^. This area has been a particular challenge for low resolution climate models to simulate properly as the models tend to produce a Gulf Stream that remains ‘attached’ to the boundary beyond the observed detachment point^15^.

On its approach to Cape Hatteras from the Tail of the Grand Banks (i.e., ‘upstream’ of the Cape in a DWBC sense), the DWBC generally lies onshore of the Gulf Stream^16^. In this region, the DWBC comprises Upper- and Classical-Labrador Sea Water (ULSW and CLSW)—formed by deep convection in the Labrador Sea—and the denser (and deeper) Overflow Waters formed as Nordic Sea waters spill over the Greenland-Iceland-Scotland sill into the North Atlantic—with these water masses splayed across the slope between the 1000-m and 4000-m isobaths, with the lighter water masses found progressively more onshore and over shallower isobaths. A ten-year record from moorings along Line W south of New England, shows strong transport variability in each of these DWBC components^17^ as well as low-frequency property variations in the LSW that can be tied to changes further upstream in the source region^9^. How these transport and property variations are communicated beyond Cape Hatteras and into to the subtropical gyre (and the role of interior versus boundary pathways in communicating variability between the gyres) remains an area of active research^18^. Tracer studies suggest that there must be a DWBC “fast track” that allows some young ULSW (recently ventilated waters with high chlorofluorocarbon concentrations) to flow along the boundary and reach 26°N with little dilution by older interior waters^7^.

Different models and observational techniques have led to different conclusions about how susceptible each DWBC layer is to the detrainment and exchange processes within the crossover region at Cape Hatteras^11,19,20^. Some of the deepest DWBC waters are observed to cross under the Gulf Stream along the boundary here^10^, but as it approaches the crossover region some water is also clearly detrained from the DWBC and flows into the abyssal interior^11,20,21^. The new observations presented here suggest that some of the lighter DWBC water masses may cross under the Gulf Stream along the boundary more readily than suggested by previous observational studies, which suggested that the lighter DWBC water masses are detrained from the boundary near the Cape.

In one of these previous observational studies, six isopycnal floats from the BOUNCE program in 1994–1995 were deployed within the ULSW layer (~800-m depth) northeast (upstream in a DWBC sense) of Cape Hatteras. These flowed along the boundary towards the crossover region near the Cape^21^. However, none continued to remain on the boundary within the DWBC past the Cape. Rather all were entrained into the separating Gulf Stream and advected eastward away from the boundary. Another study used tracer data and hydrographic sections that were relatively widely spaced around Cape Hatteras. From these, it was surmised^11^ that none of the shallowest Labrador Sea water (27.40 kg/m^3^ < σ_0_ < 27.73 kg/m^3^) passes under the Gulf Stream and that only the onshore-most portion of the deeper Labrador Sea water (27.73 kg/m^3^ < σ_0_ < 27.77 kg/m^3^) flows equatorward past Cape Hatteras in a DWBC layer that is continuous around the Gulf Stream’s separation point; the offshore portion of this layer is completely “peeled off” into the interior.

An ongoing study (PEACH: **P**rocesses Driving **E**xchange **a**t **C**ape **H**atteras, funded by the National Science Foundation) is examining the exchange of waters between the continental shelf and open ocean at Cape Hatteras including the influence of the Gulf Stream’s position and strength on the export of shelf waters. Though the DWBC is not a specific focus of PEACH, a cruise in support of PEACH on the *R/V Neil Armstrong* sampled through the DWBC in April 2017. The cruise spanned the 17-m to 3500-m isobaths on the Middle Atlantic Bight and South Atlantic Bight shelves and the adjacent deeper ocean, where the Gulf Stream transitions from an attached boundary current to a separated flow and encounters the DWBC. Here we report on six Gulf Stream crossings made during this cruise using shipboard acoustic current Doppler profilers (ADCPs). We examine the spatial evolution of the Gulf Stream and a deep counterflow observed in the velocity cross-sections. These velocity data together with observations from conductivity-temperature-depth (CTD) profiles, Argo floats, and satellite altimetry, indicate that there is a continuous path by which even the lightest DWBC water, ULSW, can cross under the Gulf Stream at Cape Hatteras instead of being pulled into the interior. Contrary to the previous studies described above, during our observation period this ‘fast track’ along the boundary was not limited to the most onshore nor the deepest part of the ULSW layer but comprised the entire ULSW layer and at least the top of the CLSW layer.

## Data

### Shipboard ADCP and CTD

To measure horizontal velocities over a range of depths and vertical resolutions, the *R/V Neil Armstrong* is equipped with shipboard ADCP sonars operating at three frequencies: a Workhorse 300 kHz (WH300), an Ocean Surveyor 150 kHz (OS150) and an Ocean Surveyor 38 kHz (OS38). Vertical and temporal resolutions of the units were set to 2-m bins over 2 minute intervals for the WH300, 4-m bins over 5 minute intervals for the OS150 and 24-m bins over 5 minute intervals for the OS38 with the shallowest bins centered at 10-m, 14-m and 44-m depth, respectively. Concurrently, the ship’s 12 kHz Knudsen echo sounder was run to obtain the collocated bottom depths.

**Figure 2:**
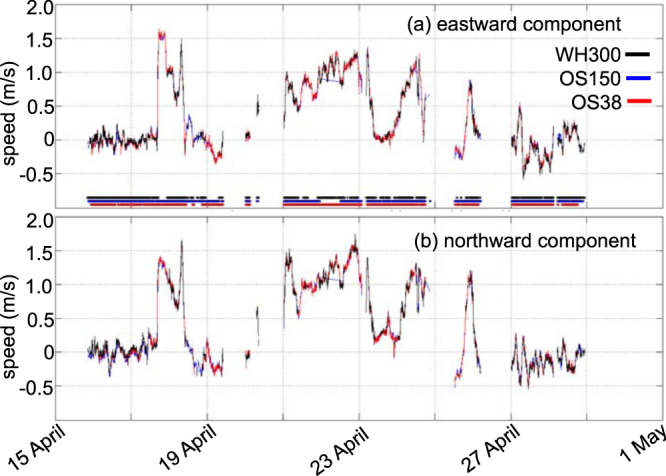
Time series of eastward (**a**) and northward (**b**) velocity components for the sonars during the entire AR-15 cruise for the WH300 (black), OS150 (blue), and OS38 (blue). Horizontal lines on (**a**) indicate the times when the respective ADCP units were returning high quality velocity data.

At depths for which there is overlap between the sonars’ bins, the agreement in the measured velocities is excellent. An example is velocity at 68-m depth (Fig. 2), shown for the entire AR-15 cruise (not just the 6 Gulf Stream crossings discussed here). Further details and plots showing the ADCP data from the individual sonars are available at http://science.whoi.edu/users/seasoar/peachadcp/. The velocities associated with the barotropic tides are generally weak in the open ocean. To confirm that tidal velocities over the continental slope and abyss off Cape Hatteras are small, we ran the ADCIRC model^22^ for the southernmost ADCP section (GS_i_). Since the tidal contribution to the velocities here is less than 0.1 cm/s (figure not shown), we did not detide the ADCP data for the Gulf Stream sections.

The 38 kHz unit returned high-quality velocity profiles reaching well below the Gulf Stream jet to maximum depths of about 1500-m or to 85% of the full water depth (strong bottom echoes contaminate the deeper bins). Since the approximate bounding depths for the ULSW layer are 750 to 1160 m—with the CLSW layer below this reaching to about 1990 m^17^, the shipboard ADCP observations can, in principle, span the whole ULSW layer and top of the CLSW layer. This is confirmed (see below) with hydrographic observations.

Six closely spaced Gulf Stream crossings were conducted between 17 and 25 April 2017 during cruise AR-15 (Fig. 1). Three were continuous sections (*GS*_*i*_, *GS*_*iv*_ and *GS*_*vi*_), each completed within 3 to 8 hours. The other sections (*GS*_*ii*_, *GS*_*iii*_ and *GS*_*v*_) were interrupted for full-water-column CTD casts measured with a Sea-Bird Electronics SBE 911plus/917plus CTD, with the deepest reaching 3508 dbar and, in the case of *GS*_*v*_, to deploy moorings. These sections took 10 to 17 hours to complete except for the most offshore cast on section *GS*_*v*_, which was taken six days after the rest of that section. *GS*_*iii*_ repeated a section collected in 2016 on the *Armstrong’s* first Science Verification Cruise (SVC1) during which evidence of the equatorward flow beneath the Gulf Stream was first noted with the OS38. *GS*_*iii*_ is also an extension of a repeat-line along which velocity data have been collected through a companion project (State of North Carolina Renewable Ocean Energy Program: Gulf Stream Observing Group) from a small boat equipped with a 300 kHz ADCP.

### Argo floats

Four SOLO-II Argo floats (MRV Systems) prepared by the Woods Hole Oceanographic Institution Argo Group were deployed during AR-15. These floats are programmed to initially sample about once per day before beginning their regular 9.9-day mission schedule. Two floats were deployed along *GS*_*v*_ and one was deployed along *GS*_*iv*_. These were rapidly advected downstream, presumably caught in the deep-reaching Gulf Stream’s poleward flow and are not considered further here. The forth float (SN 7424) was deployed along *GS*_*iii*_, and was first advected downstream by the Gulf Stream during its initial daily sampling schedule (six profiles, green dots in Fig. 1). Thereafter this float was caught in equatorward flow for several of its regular 9.9-day cycles. We argue here that this equatorward flow is the upper part of the DWBC and that this flow can round the Cape, crossing under the Gulf Stream while remaining along the boundary.

### Satellite altimetry

Mapped absolute dynamic topography at 1⁄4° resolution produced from the quality-controlled delayed-time altimetry product, is available through Aviso at daily intervals. Daily maps were averaged to produce monthly SSH maps from 1993 to the end of 2014^4^. Then for each month, the monthly-mean Gulf Stream path is taken to be the 25-cm sea surface height (SSH) contour (Fig. 1, gray curves). Since the delayed-time product is released only at 6-month intervals, the near-real time, daily product is used here to establish the position of the Gulf Stream during the AR-15 cruise (Fig. 1, red curve). From this comparison, it is apparent that the Gulf Stream was approaching the separation point in a relatively on-shore position during the cruise period. To provide additional spatial context for the ADCP sections, we also show the mapped absolute dynamic topography from the time period of the cruise (Fig. 1d).

## Results

Using the data described above, we consider the spatial evolution of the velocity structure and transport of the Gulf Stream and the upper part of an observed deep counterflow where these currents navigate one another and the bathymetry around Cape Hatteras.

### Spatial evolution of the Gulf Stream

To examine the Gulf Stream, the “poleward” direction is defined here by each section’s maximum surface velocity vector. Rotating the ADCP-measured velocity vectors to give downstream (*v*_rot_) and cross-stream (*u*_rot_) components and integrating over just the areas of positive *v*_rot_ in each section gives that section’s total poleward (Gulf Stream) transport.

During our measurement period, poleward transport increased downstream by more than a factor of two over a downstream distance of only 150 km spanning Cape Hatteras (Table 1). Though this downstream Gulf Stream intensification is consistent with previous studies^23^, these transport values may not represent the “throughput” since the sections did not reach offshore to a 0 m s^−1^ isotach. In addition, it is generally difficult to separate transports into the throughput and local recirculations associated with mesoscale eddies that impinge on the offshore edge of the Gulf Stream.

**Table 1:** Velocities and Transports at the Gulf Stream ADCP sections.

	Maximum v_rot_^1^ (m/s)	Orienta-tion (°)	Isobath^2^ (m)	Poleward transp.^3^ (Sv)	Maximum v_eq_ (m/s)	Equatorward transp.^4^ (Sv)	Duration (hrs)	Distance (km)	Start time UTC (d hr:min)
*GS*_*i*_	1.37 (1.57)	39 (42)	250 (1750)	27.8	0.25	−3.24	3.3	53	21 10:26
*Gs*_*ii*_	1.82	30	330	32.2	0.31	−4.31	10.4	50	20 23:56
*Gs*_*iii*_	1.92	46	210	33.2	0.24	−2.52	16.2	66	24 12:22
*GS*_*iv*_	1.99	45	—	58.6	0.26	−1.9	4.75	107	23 04:42
*GS*_*v*_	2.52	55	2300	74.9	0.18	−1.94	16.5	115	17 12:11
*GS*_*vi*_	2.41	39	2390	65.2	0.44 (0.14)	−3.26	8	123	18 04:41

^1^Maximum velocity observed in the upper-most bin, which is also usually the overall maximum. For GSi, the overall maximum, which is also listed (in parentheses), is at 146 m depth.

^2^Depth over which the maximum *v*_*rot*_ is found with data from the ship’s 12 kHz echo sounder (except for GS_iv_ when the data were noisy).

^3^Defined here by the direction of the maxium near-surface velocity.

^4^Defined by the local orientation of the 1000-m isobath: 30° for GS_i_ through GS_iv_ and 0° for GS_v_ and GS_vi_ with the deep-ocean maximum for GS_vi_ given in parentheses.

Notably, our April 2017 velocity sections also indicate that Gulf Stream speed increased downstream as the current flowed into deeper water. At the southernmost section, *GS*_*i*_, the maximum near-surface velocity is 1.4 m/s. At *GS*_*iv*_ the maximum is 2.0 m/s and at the northernmost sections (*GS*_*v*_ and *GS*_*vi*_) the speeds reach ~2.5 m/s. For the three southern sections—*GS*_*i*_ through *GS*_*iii*_, all south of the “separation point”—the location of the maximum surface velocity vector falls between the 210-m and 330-m isobaths. For the “separated Gulf Stream” (i.e., at sections *GS*_*v*_ and *GS*_*vi*_) the maximum surface velocities are found over the 2300 to 2400-m isobaths. Such a local and intense acceleration around Cape Hatteras has not been previously reported for Eulerian nor stream-coordinates time-averages, but is consistent with a 2014 snapshot from quality-controlled CODAR radar data^24^.

**Figure 3:**
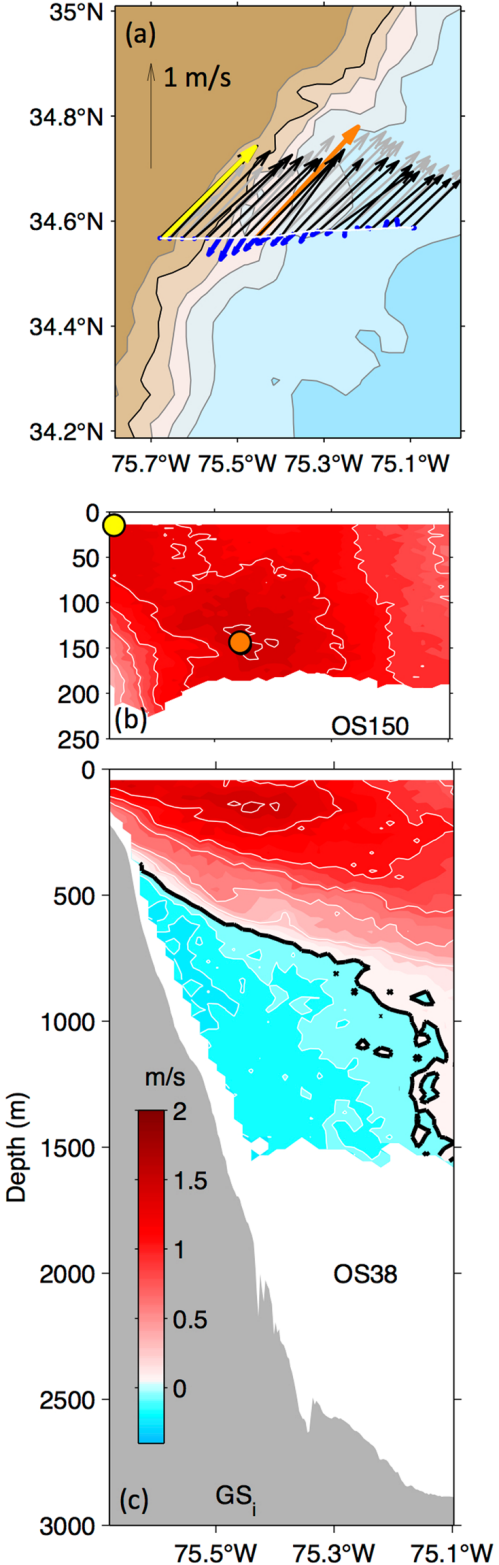
Velocities at *GS*_*i*_. Panel (a) plan view near-surface (black vectors with maximum highlighted in yellow), at 146-m depth (grey with the section’s overall maximum highlighted in orange) and at 980-m depth (blue). Isobaths contoured at 500-m increment (gray) with 1000-m isobath highlighted (black). Panel (b) poleward velocity (*v*_*rot*_, towards 39°) from the OS150. Yellow and orange dots correspond to the colored vectors (a). Panel (c): poleward velocity (*v*_*rot*_) from the OS38 with bottom topography from the ship’s underway 12 kHz echo sounder. Black line highlights the 0 m/s isotach. Contour intervals, shown in white, for positive velocities in (b) and (**c**) are 0.25 m/s and 0.10 m/s for negative velocities. The map in (a) was generated using Matlab 2014b (Mathworks, Inc.).

Locations of the near-surface velocity maxima observed with the ADCPs agree remarkably well with the position of the Gulf Stream axis inferred from the 25-cm SSH contour (compare the red curve and yellow dots in Fig. 1). At most sections, Gulf Stream speed is highest in the shallowest ADCP bin (centered at 14-m depth for the OS150). The exception is at *GS*_*i*_ (Fig. 3). Here the near-surface flow (1.4 m/s over the 250-m isobath, yellow vector) is not the strongest; rather the highest speed (1.6 m/s over the 1750-m isobath, orange vector) is found at 146-m depth. As expected, the 25-cm contour tracks the location of the near-surface maximum, not this subsurface maximum (compare the red line and orange dot at *GS*_*i*_ in Fig. 1).

### Spatial evolution of the counterflow

**Figure 4:**
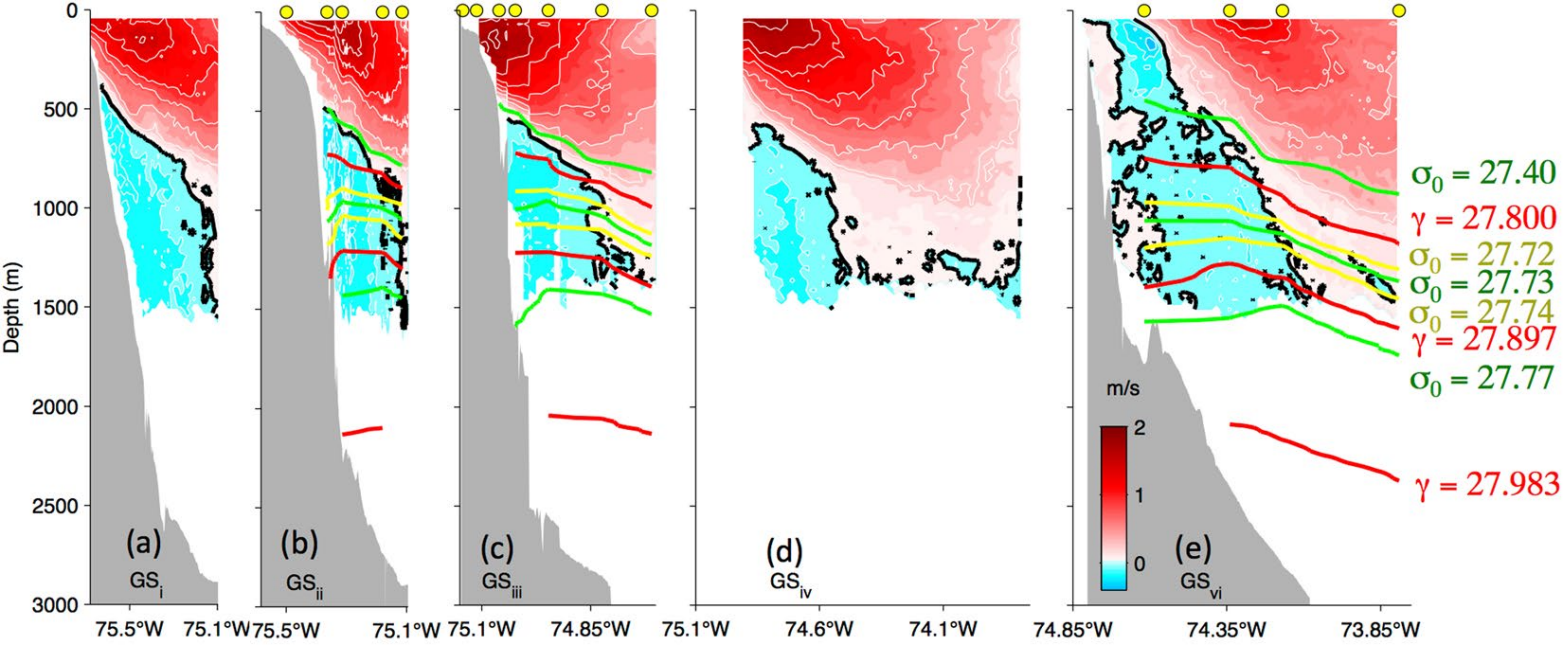
Velocities measured by the OS38 (shaded) rotated to highlight equatorward flow (*v*_*eq*_) across each section with the 0 m/s isotach (black) highlighted and positive velocities (towards 30° for a-d and northward for e) and negative (equatorward) velocities contoured at 0.25 m/s and 0.10 m/s intervals, respectively (white). Green, red and yellow curves highlight isopycnals (kg/m^3^) determined from the full-water column CTD casts (locations indicated with yellow dots) taken during the respective ADCP sections. (At *GS*_*vi*_ the isopycnals are calculated from casts taken along the same track over the previous 24 hours during crossing *GS*_*v*_).

The ADCP data show a deep counterflow beneath the onshore edge of the poleward-flowing Gulf Stream across each of the six velocity sections (Fig. 4). The orientation of the 1000-m isobath, which curves sharply around the Cape, is used to define “equatorward” flow (*v*_*eq*_) at each section. In the sections spanning the separated Gulf Stream (*GS*_*v*_ and *GS*_*vi*_), this isobath is roughly meridional and equatorward flow is simply southward (*v*_*eq*_ = −*v*, towards 180°). Here *v*_*eq*_ is generally less than 10 cm/s (except for a narrow upper-ocean feature, centered at 134-m depth, where *v*_*eq*_ reaches 44 cm/s in *GS*_*vi*_). The deep equatorward speeds are consistent with near-bottom DWBC flows observed during the SYNOP program (Fig. 1, blue arrows), where the 7-month means observed between the 2000-m and 4000-m isobaths were about 5 cm/s^5^. Despite relatively low speeds, net equatorward transport captured by the OS38 is substantial since the equatorward flow covers a large cross-sectional area: 1.9 Sv for *GS*_*v*_ and 3.2 Sv for *GS*_*vi*_ (Table 1).

South of 35.5°N the 1000-m isobath runs towards 30° and *v*_*eq*_ is towards 210°. Once the counterflow rounds the Cape and is squeezed between the Gulf Stream and the steep continental slope here, *v*_*eq*_ intensifies. Equatorward speeds at these sections (*GS*_*i*_ through *GS*_*iv*_) exceed 20 cm/s and equatorward transport (integrated where *v*_*eq*_ > 0) is about 2-3 Sv (Table 1). Since the shipboard ADCP could not measure the deepest velocities (both due to strong interference from bottom returns over the slope and because waters at depths greater than 1500 m are beyond the reach of the OS38), these values represent a minimum bound on the equatorward transport.

## Discussion

**Figure 5:**
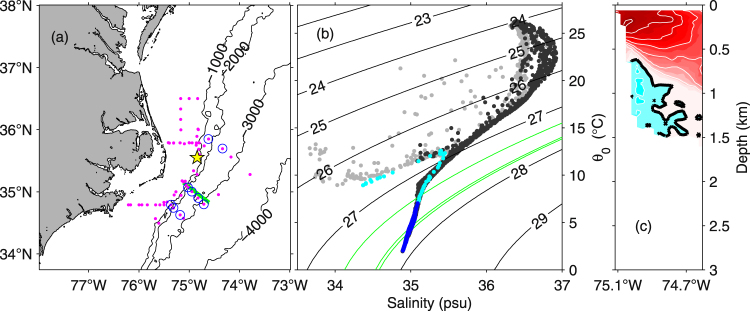
Panel (a) map of CTDs from AR-15 (magenta) with those circled at which equatorward deep flow was observed (blue). Also shown is ‘the Point’ by Hatteras (star), and location of the SVC1 ADCP section (green). Panel (b) theta-S curves for casts over isobaths shallower than 500-m (light gray) and exceeding 500-m (black, blue and cyan) where the blue and cyan dots highlight the portions of these deep-reaching casts where equatorward flow was observed (Fig. 4). The upper 500 m of the cast which sampled through the strong upper-ocean feature noted in section *GS*_*vi*_ (Fig. 4e) is shown in cyan. Black lines show σ_0_ (kg/m^3^) with green lines highlighting shallow (27.40 kg/m^3^ < σ_0_ < 27.73 kg/m^3^) and deeper (27.73 kg/m^3^ < σ_0_ < 27.77 kg/m^3^) Labrador Sea Water classes of^11^. The σ_0_ = 27.72 kg/m^3^ and 27.74 kg/m^3^ (yellow lines in Fig. 4) fall almost on top of the central (27.73 = σ_0_) green line. Panel (c) *v*_*eq*_ from SVC1 (along green line in a), with shading and contours as in Fig. 4. The map in (a) was generated using Matlab 2014b (Mathworks, Inc.).

CTD casts (Fig. 5) suggest that the observed counterflow is the upper part of the equatorward-flowing DWBC. Using water mass definitions based on neutral density, γ,^6^ from Toole *et al*.^16^ the sections’ ULSW (27.800 kg/m^3^< γ < 27.897 kg/m^3^) and CLSW (27.897 kg/m^3^ < γ < 27.983 kg/m^3^) are indicated by the red curves in Fig. 4. These isopycnals slope across the sections and are deeper within the deep-reaching Gulf Stream on the offshore sides of the sections. On the onshore sides—where the counterflow is observed—the OS38 captures the entire ULSW layer and even the upper-most CLSW.

In Pickart and Smethie^11^ water masses were considered using potential density referenced to the surface, σ_0_ (green curves in Figs 4 and 5). They found that none of the lightest Labrador Sea Water (the layer between the widely-spaced green curves on the theta-S plot in Fig. 5b) and only some of the ‘intermediate’ Labrador Sea Water (between the tightly spaced green curves in Fig. 5b) rounds Cape Hatteras along the boundary. In contrast, our shipboard observations from AR-15 show equatorward flow in *both* density classes (blue dots in Fig. 4b).

Another set of isopycnals shown in Fig. 4 (yellow) depicts the density range within which RAFOS floats were deployed in the BOUNCE program. These floats were ejected in the crossover region^21^. In contrast, the AR-15 velocity sections examined here suggest a smooth, connected flow in this density class. Indeed this impression of a “connected” flow is corroborated by the trajectory of the Argo float which was entrained in the deep flow, nominally at 1000 dbar from the 6^th^ to the 8^th^ satellite fix (Fig. 1a, green dots). With about 10 days between these fixes and 230 km covered by the float, the mean speed experienced by the float in this layer is 27 cm/s, which is remarkably close to the maximum *v*_*eq*_ from the ADCP velocity sections (Table 1).

Finally, to provide context for these “snapshot” sections, we consider the 10-year mean equatorward transport observed by the Line W mooring array across the DWBC south of Cape Cod (Fig. 1d, yellow line). Here the mean is 4.33 ± 0.46 Sv for the ULSW layer and 7.37 ± 1.20 Sv for the CLSW layer^17^ (reporting their ‘average of daily profiles’). Not surprisingly, the AR-15 transports are somewhat lower (1.9–4.3 Sv, Table 1). This may be due in part to the limited reach of the OS38 over topography and may reflect detrainment of some DWBC between Line W and Cape Hatteras (particularly in the ULSW layer).

## Conclusions

Shipboard observations from AR-15 and the Argo float deployed during that cruise, suggest that flow in the DWBC’s ULSW and CLSW layers is continuous along the boundary around the Cape, at least under some circumstances. This is corroborated by the SVC1 cruise in 2016 when a deep counterflow was also observed with the OS38 in the section along *GS*_*iii*_ (Fig. 5c). Shipboard data from upcoming cruises and mooring data presently being collected through the PEACH program (https://sites.google.com/a/ncsu.edu/peach-public-site/home) will help characterize variability in this crossover region and will provide context for the data presented here so that the underlying dynamics can be investigated further.

Combining shipboard ADCP sonars operating at different frequencies provides a powerful observational tool to characterize the ocean. The 38 kHz unit is making observations of the deep ocean more readily accessible and useful datasets are being built up by the now-routine sampling from vessels in the UNOLS fleet like the *R/V Neil Armstrong*. In addition to the research fleet, ships of opportunity are also collaborating with the research community to outfit commercial vessels with ADCPs^25,26^. Efforts are underway to equip the *C/V Oleander*, a container ship that makes weekly round trips between New Jersey and Bermuda, crossing the shelf flow, DWBC, Gulf Stream and Sargasso Sea (green track in Fig. 1d), with a 38 kHz ADCP sonar. The data presented here from the *R/V Neil Armstrong* suggest that the *C/V Oleander’s* crossings with the OS38 will enable regular sampling not only of the deep-reaching Gulf Stream, but also of the upper part of the DWBC, providing valuable observations for studies of the AMOC’s cold and warm limb flows.

### Data availability

Data are available through http://marine.copernicus.eu (altimetry) and http://argo.whoi.edu (Argo) and http://science.whoi.edu/users/seasoar/peachadcp/.
